# Differences in gene regulation by TLR3 and IPS-1 signaling in murine corneal epithelial cells

**DOI:** 10.1038/s41598-023-35144-1

**Published:** 2023-05-16

**Authors:** Seitaro Komai, Mayumi Ueta, Hiromi Nishigaki, Katsura Mizushima, Yuji Naito, Shigeru Kinoshita, Chie Sotozono

**Affiliations:** 1 Department of Ophthalmology, Kyoto Prefectural University of Medicine, 465 Kajii-Cho, Kamigyo-Ku, Kyoto, Japan; 2 Department of Human Immunology and Nutrition Science, Graduate School of Medical Science, Kyoto Prefectural University of Medicine, Kyoto, Japan; 3 Department of Frontier Medical Science and Technology for Ophthalmology, Kyoto Prefectural University of Medicine, Kyoto, Japan

**Keywords:** Innate immunity, Gene expression, Gene regulation, Mucosal immunology, RIG-I-like receptors, Toll-like receptors

## Abstract

Toll-like receptor 3 (TLR3) and interferon-beta promoter stimulator-1 (IPS-1) are associated with antiviral responses to double-stranded RNA viruses and contribute to innate immunity. We previously reported that conjunctival epithelial cell (CEC) TLR3 and IPS-1 pathways respond to the common ligand polyinosinic:polycytidylic acid (polyI:C) to regulate different gene expression patterns as well as CD11c + cell migration in murine-model corneas. However, the differences in the functions and the roles of TLR3 and IPS-1 remain unclear. In this study, we investigated the differences of TLR3 or IPS-1-induced gene expression in corneal epithelial cells (CECs) in response to polyI:C stimulation using cultured murine primary CECs (mPCECs) derived from TLR3 and IPS-1 knockout mice via comprehensive analysis. The genes associated with viral responses were upregulated in the wild-type mice mPCECs after polyI:C stimulation. Among these genes, Neurl3, Irg1, and LIPG were dominantly regulated by TLR3, while interleukin (IL)-6 and IL-15 were dominantly regulated by IPS-1. CCL5, CXCL10, OAS2, Slfn4, TRIM30α, and Gbp9 were complementarily regulated by both TLR3 and IPS-1. Our findings suggest that CECs may contribute to immune responses and that TLR3 and IPS-1 possibly have different functions in the corneal innate immune response.

## Introduction

Toll-like receptor 3 (TLR3) is a pattern recognition receptor that recognizes double-stranded RNA (dsRNA) and contributes to antiviral responses in the innate immune system by recognizing dsRNA and producing interferon (IFN) and other cytokines^1^. TLR3 is expressed on endosomes of immune cells such as dendritic cells and macrophages^1^, and on the cell surface of some kinds of epithelial and vascular endothelial cells^2–4^. We have previously reported that TLR3 is a disease-associated gene in Stevens-Johnson syndrome (SJS), and there is a possibility that an abnormality in TLR3-related innate immune mechanisms is relevant to the pathogenesis of ocular surface inflammation in SJS^5^. Understanding of the mechanisms to regulate immune responses by TLR3 signaling which could be activated by dsRNA may give us a better understanding of the pathophysiology of SJS.

We also previously reported that TLR3 is particularly strongly expressed in human corneal and conjunctival epithelial cells compared to human peripheral (blood) mononuclear cells, and that TLR3 is expressed on the cell surface^6,7^. Cultured human corneal cells treated with viral dsRNA mimicking *polyinosinic:polycytidylic* acid (polyI:C) showed increased expression of cytokines (IL-6 and IL-8)^7^, and chemokines (CCL5, CCL20, CXCL10, and CXCL11)^8^. At the same time, their response to lipopolysaccharide (LPS) was poor^7^. For innate immunity against dsRNA viruses, it is necessary to consider that the intracellular receptors *melanoma differentiation-associated gene 5* (MDA5) and *retinoic acid-inducible gene-I* (RIG-I) respond to dsRNA as well as TLR3^1^. MDA5 is involved in type 1 IFN production, TLR3 contributes to IL-12p40 synthesis, and dendritic cells require both receptors to produce IL-6^9^. Moreover, literature shows that MDA5 and TLR3 have complementary roles in natural killer (NK) cell activation^10^, and that TLR3 and RIG-I have different cell-specific dominance levels in antiviral responses^11^. However, the different roles of the TLR3 and RIG-I/MDA5 pathways remain unclear.

Our past research demonstrated differential regulation of gene expression by poly I:C stimulation in murine conjunctival epithelium using mice with knockout (KO) of IFN-beta promoter stimulator 1 (IPS-1, also known as MAVS, VISA, and Cardif), an adaptor protein for RIG-I, MDA5, and TLR3 KO mice. The findings in that study revealed that chemokines such as CXCL10 and CCL5 are predominantly regulated by IPS-1 and TLR3, respectively, and that gene expression associated with several immune responses is differentially regulated by TLR3 and IPS-1^12^. Upon further study, we found that CD11c + cells infiltrate the central cornea in response to polyI:C stimulation, and that TLR3 KO and IPS-1 KO mice showed different distributions and kinetics of CD11c + cells in the cornea^13^. Based on these data, we speculated that TLR3 and IPS-1 signaling differentially regulate CD11c + cells. Our findings suggest that TLR3 and IPS-1 signaling may play distinct roles in corneal epithelial cells (CECs).

In the present study, we analyzed the changes in gene profiles of murine primary CECs (mPCECs) stimulated with polyI:C using comprehensive gene analysis to elucidate the differential TLR3 and IPS-1 regulation of genes associated with immune responses in CECs.

## Results

### Changes in gene expression in wild-type (WT) murine CECs in response to polyI:C stimulation

**Figure 1:**
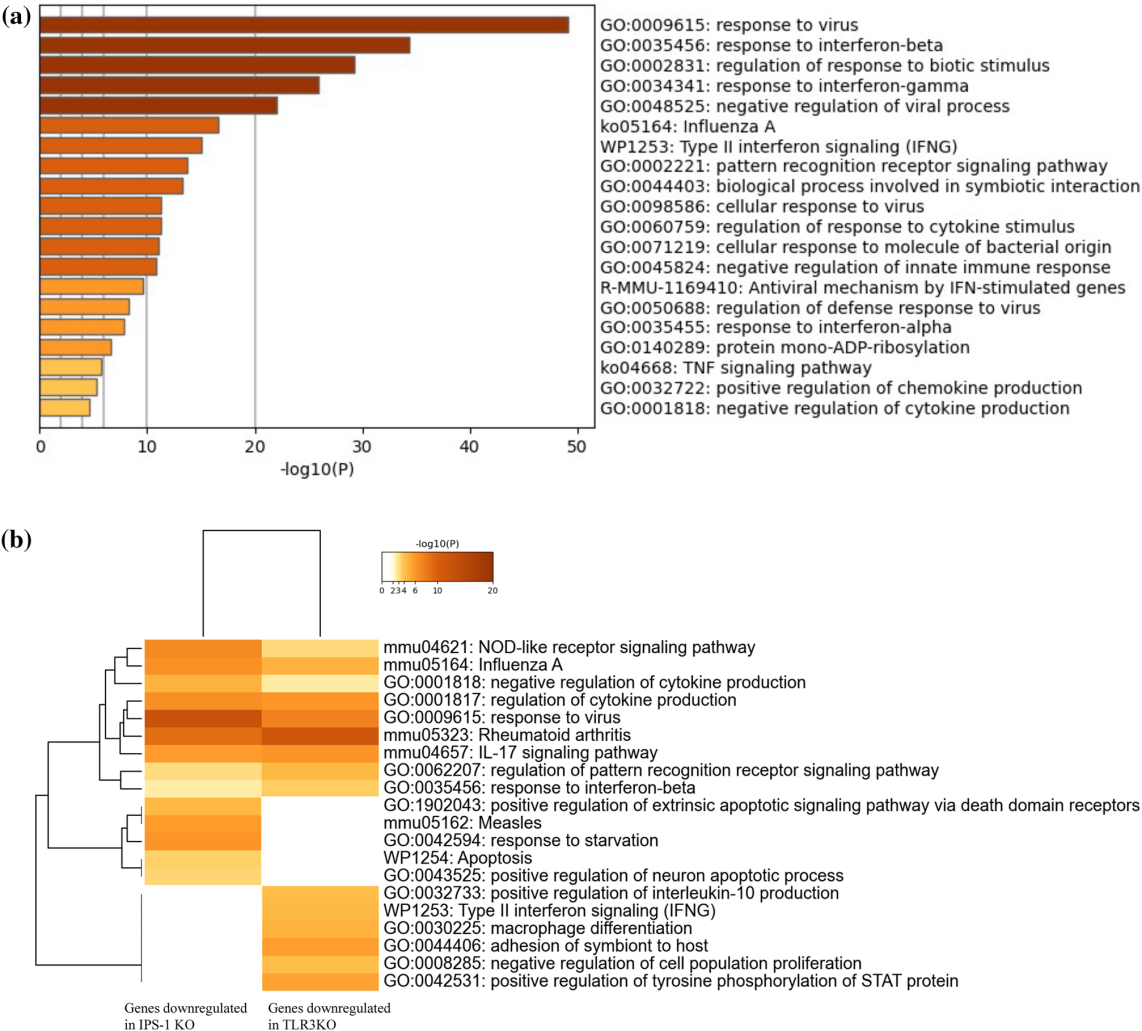
Gene Ontology (GO) analysis of genes induced in murine corneal epithelial cells (CECs) by polyinosinic:polycytidylic acid (polyI:C) stimulation. (**a**) GO analysis of 121 genes upregulated in cultured murine primary CECs (mPCECs) derived from wild-type (WT) mice after 6 h of culture with 10 μg/ml polyI:C. (**b**) Results of GO analysis of genes downregulated in mPCECs derived from Toll-like receptor 3 (TLR3) knockout (KO) and interferon-beta promoter stimulator 1 (IPS-1), with the KO mice compared to WT mPCECs after polyI:C stimulation. GO analysis was performed and figures were obtained using Metascape (open-access resource).

To investigate the response of murine CECs to polyI:C stimulation, we performed a comprehensive gene analysis of mPCECs. mPCECs derived from WT, TLR3 KO, and IPS-1 KO mice. These were treated with polyI:C, and then analyzed for changes in gene expression with and without stimulation using GeneChip® (Affymetrix, Inc., Santa Clara, CA) analysis. In the WT mPCECs, polyI:C stimulation resulted in a more than twofold increase in the expression of 121 genes (Supplementary Table 1), while the expression of 4 genes decreased to less than 50%. Gene Ontology (GO) analysis of those 121 upregulated genes revealed that many were related to viral responses and responses to IFN (Fig. 1a). Next, we compared the gene expression of WT polyI:C-stimulated mPCECs to that of TLR3 KO mice and found that the expression of 43 genes decreased to < 50% in the TLR3 KO. Similarly, in the IPS-1 KO, 116 genes were downregulated to < 50% compared to the WT (Supplementary Table 2). GO analysis of these genes showed that they were related to viral responses, rheumatoid arthritis, IL-17 signaling, and the regulation of cytokine production (Fig. 1b).

Thus, our results suggest that when stimulating the cornea with polyI:C, CECs and immune cells demonstrate an antiviral response, with both TLR3 and IPS-1 signals involved in the process.

### Involvement of TLR3 and IPS-1 in cytokine/chemokine production in mPCECs

**Figure 2:**
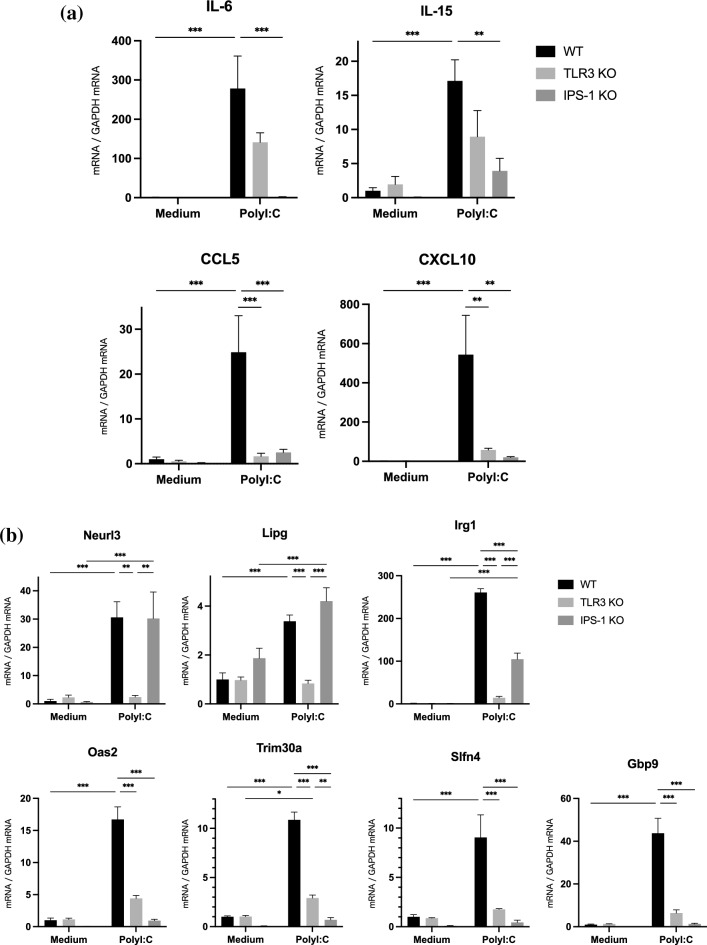
Genes associated with immune and/or inflammatory responses induced by polyI:C downregulated by KO of TLR3 and IPS-1 in cultured mPCECs. (**a**) Gene expression of 4 cytokines/chemokines and (**b**) gene expression of 7 proteins whose expression was increased in WT mPCECs upon polyI:C stimulation. Gene expression levels of WT, TLR3 KO, and IPS-1 KO mPCECs with or without polyI:C stimulation were measured by quantitative polymerase chain reaction. The data are representative of 3 independent experiments. and presented as the mean SEM of 6 samples (**P < 0.01, ***P < 0.001, ns = not significant; one-way ANOVA with Tukey's multiple comparisons test).

To investigate the effects of TLR3 and IPS-1 KO on cytokine and chemokine production, we screened for cytokines and chemokines in the 121 genes upregulated by polyI:C stimulation in WT mPCECs. IL-6, IL-15, CCL5, and CXCL10 were screened from the results of GeneChip® gene expression analysis. While the mRNA of CCL5 and CXCL10 was significantly downregulated in both TLR3 KO and IPS-1 KO mPCECs compared to WT, IL-6 and IL-15 showed significant downregulation only in the IPS-1 KO mPCECs (Fig. 2a).

### Differential roles of TLR3 and IPS-1 signaling pathways in response to polyI:C stimulation

To find genes regulated explicitly by the TLR3 and IPS-1 pathways (i.e., RIG-I, MDA5) that recognize the same ligand, we compared the gene expression profiles of mPCECs from TLR3 KO and IPS-1 KO mice. Among the 121 genes upregulated by polyI:C stimulation in WT mPCECs, we screened genes with more than a twofold difference in expression between TLR3 KO and IPS-1 KO after polyI:C stimulation. The gene expression levels of Neurl3, *immune-responsive gene 1* (Irg1), and *endothelial type lipase G* (LIPG) were less than half in TLR3 KO compared to IPS-1 KO. On the other hand, expression levels of *2′,5′-oligoadenylate synthase* (OAS) 2 (OAS2), *Schlafen4* (Slfn4), *E3 ubiquitin ligase tripartite motif protein 30α* (TRIM30α), and *guanylate-binding protein 9* (Gbp9) were less than half in IPS-1 KO compared to TLR3 KO.

Next, we measured the expression levels of those genes by quantitative reverse transcription polymerase chain reaction (RT-qPCR). Neurl3 and LIPG were significantly downregulated only in TLR3 KO mPCECs. In addition, we observed Irg1 downregulation in both the TLR3 KO and IPS-1 KO mPCECs, and we also found a significant difference in the TLR3 KO mPCECs compared to that of the IPS-1 KO mPCECs. These findings suggest that TLR3 is dominantly responsible for regulating the expression of those three genes (Fig. 2b). OAS2, Slfn4, TRIM30αa, and Gbp9 were also downregulated in both TLR3 KO and IPS-1 KO mPCECs. Still, the downregulation was more pronounced in the IPS-1 KO mPCECs (Fig. 2b).

These results indicate that the TLR3 and IPS-1 pathways regulate different genes in response to the same ligand: polyI:C.

## Discussion

This comprehensive genetic analysis study of cultured mPCECs revealed that the genes related to the antiviral response are upregulated by polyI:C stimulation, and that the TLR3 and IPS-1 (RIG-I or/and MDA5) signaling pathways are involved in this response. Furthermore, the expression profiles also revealed some differences in the roles of the TLR3 and IPS-1 signaling pathways, for which polyI:C is the common ligand.

In the GO analysis, both in TLR3KO and IPS-1KO, cytokine production and viral defense pathways were downregulated, as well as the IL-17 signaling pathway and the Nod-like receptor signaling pathway. The IL-17 signaling pathway recruits TRAF6 to activate downstream NF-kB and MAPK, as well as TLR3 and IPS-1 signaling. Furthermore, the NOD-like receptor (NLR) signaling pathway is also common to TLR3 and IPS-1 in that it activates IKKα, IKKβ, and IKKγ (NEMO) followed by NF-kB activation^1,14,15^. Therefore, the functions downregulated in corneal epithelial cells derived from TLR3KO and IPS-1KO were mainly viral response, cytokine production, and pattern recognition receptor signaling pathways. Since knockout of only one of TLR3 and IPS-1 affects antiviral activity, as expected, each of TLR3 and IPS-1 was suggested to be responsible for the innate immune response in corneal epithelial cells. On the other hand, downregulation of gene expression associated with IL-10 production, Type II IFN signaling, and macrophage differentiation was observed only in TLR3KO, suggesting that there may be different functions in the immune response between TLR3 and IPS-1 signaling.

Expression of cytokines/chemokines such as IL6-, IL-15, CCL5, and CXCL10 was upregulated by polyI:C stimulation in WT mPCECs. As for the gene expression of IL-6 and IL-15, KO of IPS-1 markedly reduced the expression of those genes, indicating that IPS-1 may predominantly contribute to the production of those cytokines in CECs. On the other hand, the expression of CCL5 and CXCL10 was decreased by KO of both TLR3 and IPS-1, thus suggesting that those genes may be cooperatively regulated by the TLR3 and IPS-1 pathways. Our previous report showed that CXCL10 and CCL5 are predominantly regulated by IPS-1 and TLR3, respectively, differing from our present results^12^. Two differences in the previous report from the present study are that it was an in vivo experiment and that different types of cells (conjunctival epithelial cells) were used. In vivo, epithelial cells are surrounded by conjunctival tissue and tear fluid, and their interaction with conjunctival tissue or immune cells could influence gene expression in epithelial cells. Furthermore, corneal and conjunctival epithelium are epithelial cells covering the ocular surface. Still, conjunctival tissue is vascular-rich and contains numerous immune cells, while corneal epithelium has no blood vessels and few immune cells. Since corneal tissue must remain transparent to maintain vision, and intense inflammation and cellular infiltration sometimes interfere with such corneal transparency, it is unsurprising that corneal and conjunctival epithelia have biologically distinct properties in the mechanisms that regulate inflammation. Therefore, these experimental approach and cell type differences may have led to different results in regulating CXCL10 and CCL5.

In a previous study, we reported that TLR3 and IPS-1 are involved in the distribution and the polyI:C-induced migration of CD11c + cells to the cornea^13^. Furthermore, IL-6 plays a vital role in innate immune response^16^, and IL-15 is involved in differentiating and maintaining T cells and NK cells^17,18^. Moreover, CCL5 and CXCL10 are present in migrating immune cells^19,20^. Therefore, differences in the expression of these chemokines and cytokines may be involved with the differential regulation of CD11c + cells in the cornea.

In addition, this study’s comprehensive genetic analysis findings also showed that the TLR3 and IPS-1 pathways might regulate the expression of distinct genes. TLR3 predominantly regulated Neurl3 and LIPG expression. TLR3 and IPS-1 complementarily regulated Irg1, yet TLR3 seemed more dominant. In terms of the mechanisms of immune signaling regulation, both TLR3 and IPS-1 promote IFN transcription following activation of IRF-3 through IKKε(IKKi)/TBK1 activation^1^. Furthermore, both are known as Interferon stimulated genes (ISGs) and constitute a network of positive feedbacks in which TLR3 and IPS-1 pathways are activated via IFNAR1^21–23^. Expression of genes whose transcription depends on this network would be compensated even if one of TLR3 or IPS-1 is knocked out in signaling against PolyI:C. It may be possible that TLR3 and IPS-1 worked complementarily, resulting in fewer genes whose expression was downregulated in TLR3KO and IPS-1KO.

On the other hand, Abe et al. reported that IRF-3 activation depends on IKKβ and IKKγ in TLR3 signaling but not RLR (IPS-1) signaling^24^. Differences in IRF-3 activation mechanisms, or other differences, may lead to the unique functions of TLR3 and IPS-1 suggested in this study. Moreover, gene expression of CCL5 and CXCL10 was strongly downregulated in both TLR3KO and IPS-1KO, which indicates that the expression of these genes requires both TLR3 and IPS-1 signaling. Since it has been previously reported that TNF-α and TLR3 signaling cooperatively regulate IRF-3^24,25^, it is suspected that an unknown cross-talking network exists and that TLR3 and IPS-1 signaling cooperatively regulate several gene expressions. However, the entire complex network involving TLR3 and IPS-1 is still unclear, and further studies are needed to elucidate the molecular mechanisms underlying the cooperative or unique functions of TLR3 and RLR (IPS-1) signaling.

Considering the functional differences in immune signaling between TLR3 and IPS-1 from uniquely expressed genes, Neurl3 is upregulated upon hepatitis C virus (HCV) infection and inhibits HCV replication. MDA5 is required for IFN signaling during HCV infection of hepatocytes, while Neurl3 expression is induced independently of IFN^26^. Therefore, Neurl3 may be produced without the MDA5 signaling pathway, and our findings revealed that TLR3 KO specifically downregulated Neurl3. This suggests that Neurl3 production may be regulated by the TLR3 signaling pathway, unrelated to MDA5 or IFN production.

LIPG is a lipoprotein produced mainly by vascular endothelial cells. It is associated with lipid metabolism and is an acute phase response reactant that mediates inflammatory processes via inflammatory cytokines^27^. Moreover, LPS stimulation upregulates LIPG expression in macrophages via TLR^28^. Hence, LIPG may reflect some type of host defense in acute or chronic inflammatory conditions. Furthermore, the present findings suggest that LIPG reflects an immune response through the TLR3 pathway since TLR3 predominantly regulates LIPG.

Irg1 is a mitochondrial enzyme that is highly expressed in macrophages during inflammation, and it produces itaconic acid, which is involved in defense against Salmonella enterica and Mycobacterium tuberculosis^29,30^. Moreover, Irg1 might have a role in regulating immune cell metabolism to minimize pathological inflammatory responses^30^. It has also been reported that Irg1 is enhanced when macrophages are stimulated with LPS but not by polyI:C stimulation^31^. Our finding that polyI:C stimulation increases the gene expression of Irg1 in CECs via a TLR3-dependent manner suggests that epithelial cells and immune cells may have different roles in innate immunity, which supports our previous reports showing that ocular surface epithelial cells are involved in the immune response^32,33^.

OAS2, Slfn4, TRIM30α, and Gbp9 were found to be downregulated in both the TLR3 KO and IPS-1 KO mPCECs. Since downregulation of gene expression was more marked in the IPS-1 KO than in the TLR3 KO, those genes may be predominantly regulated by the IPS-1 signaling pathway.

The OAS family of genes are IFN-stimulated genes and are associated with the antiviral activity of IFN^34^. OAS is a kind of ISG, and type 1 IFN enhances its production via the INFα receptor complex^34–36^. Moreover, it has been reported that OAS2 is induced in a RIG-I-dependent manner^37^.

Slfn4 is a member of the *Schlafen* gene family^38^, yet its specific function has yet to be fully elucidated. It has been reported that TLR ligands such as polyI:C and LPS increase Slfn4 expression in activated macrophages and decrease during their differentiation stages, suggesting that it may participate in myelopoiesis^39^.

TRIM30α is a member of the TRIM family, which is involved in a variety of cellular processes, including cell proliferation, differentiation, development, oncogenesis, and apoptosis^40^. TRIM30α is induced by TLR-mediated activation of the nuclear factor kappa-light-chain-enhancer of activated B cells (NF-kB), and negatively regulates NF-kB by a feedback mechanism^41^. In addition, Kumar et al. reported that NF-kB is activated by polyI:C stimulation in human corneal epithelium^42^. Therefore, the changes in TRIM30α gene expression observed in this present study may have been the result of changes in the activity of NF-kB in response to polyI:C stimulation by KO of TLR3 or IPS-1.

Gbp9 is an IFN-γ-inducible GTPase involved in immune responses against microbes and viruses. To date, there have been few reports on Gbp9, but it has been reported that it may be related to the polarization of macrophage M1 or M2^43^.

However, to our knowledge, there are limited reports on these genes and no reports on corneal tissues. It should be noted that this discussion does not concider differences between cell types, as there may be different gene regulation mechanisms of TLR3 and IPS-1 depending on the cell type.

It should be noted that the genes picked up in the present analysis were all related to immune and inflammatory responses. Therefore, this suggests that corneal epithelial cells are involved in the innate immune response on the corneal surface through TLR3 and IPS-1 signaling pathways. Furthermore, the fact that these pathways regulate different genes implies that TLR3 and IPS-1 have distinct functions in the innate immunity in the cornea. However, the various functional roles of TLR3 and IPS-1 in the immune response in the cornea are still unclear. Therefore, further investigations are needed to clarify the interaction of epithelial and immune cells. For example, the relationship between cytokines and chemokines induced by CECs and CD11c + cells in cornea.

In the present study, we analyzed the results at a time point of 6 h after PolyI:C stimulation. This is consistent with our previous report, showing that the expression of several genes was markedly upregulated in the ocular surface cells at 6 h after exposure to antigens associated with innate immunity^7,8,12,32^. We note, however, that further insights may be obtained by analyzing gene expression at different time points.

In summary, in this study, we performed a comprehensive analysis of genes using cultured mPCECs, and our findings demonstrate that CECs contribute to innate immune responses via TLR3 and IPS-1. In addition, the TLR3 and IPS-1 signaling pathways differentially regulate the expression of genes associated with immune and inflammatory responses.

## Methods

### Mice

The BALB/c background TLR3 KO mice and C57BL/6 background IPS-1 KO mice used in this study were the kind gift of Dr. Shizuo Akira (Osaka University, Osaka, Japan). The C57BL/6 background IPS-1 KO mice were backcrossed with BALB/c mice for more than 6 generations to produce the BALB/c background IPS-1 KO mice.

### mPCECs

mPCECs were cultivated and used for the GeneChip® gene expression analysis and RT-qPCR. After the mice were euthanized by intraperitoneal injections of overdose of pentobarbital, the corneas were harvested from the eyes of the mice, aged 4–6 weeks, at the time of being euthanized, and incubated in 1.2 U/ml of purified Dispase (Roche Diagnostics, Basel, Switzerland) for 18 h at 4 °C. CEC sheets were detached with forceps, and then incubated in 500 μl of 0.05% trypsin–EDTA (Life Technologies, Carlsbad, CA). The epithelial sheets from multiple eyes (at least 3 mice, 6 eyes) were mixed and separated into single cells by pipetting. To inhibit the activity of trypsin, Fetal Bovine Serum (Life Technologies) was added to the medium. Then, isolated cells were cultured in a fibroblast proliferation medium (CnT-Prime Fibroblast Proliferation Medium; CELLnTEC Advanced Cell Systems AG, Bern Switzerland) on a collagen (Cellmatrix, Type I-C; Nitta Gelatin, Inc., Osaka, Japan) coated 48-well plate (Corning, Inc., Corning, NY). The cultures were incubated at 3 °C under 95% humidity and 5% CO_2_. After reaching 80% confluence in 3–5 days, the cultured mPCECs were stimulated with or without polyI:C, and then collected. The duration of the culture of the cells stimulated with 10 μg/ml polyI:C was adjusted to 6 h to optimize the maximum induction of IFNβ, CCL5, and CXCL10 based on the result of RT-qPCR at multi-time points (Supplementary Figure 1).

## Gene expression analysis

mPCECs derived from WT, TLR3 KO, and IPS-1 KO mice were stimulated with or without 10 μg/ml of polyI:C for 6 h. To ensure a sufficient amount of RNA, 6 wells of mPCECs were collected, and then mixed together to make each sample. Total RNA was extracted using the RNeasy® Kit (Qiagen, Valencia, CA) and processed for use with Affymetrix GeneChip® Mouse Gene 1.0 ST Array (Affymetrix), with the cRNA preparation and target hybridization carried out in accordance with the manufacturer's instructions. DNA chips were scanned with a dedicated confocal scanner (GeneChip® Scanner 3000; Affymetrix). The obtained data were then exported as CEL files and analyzed using the Transcriptome Analysis Console (TAC; Thermo Fisher Scientific™, Waltham, MA). GO analysis was performed using Metascape (open-access resource available at http://metascape.org. )^44^.

### Real-time RT-qPCR

Total RNA was extracted from mPCECs using TRIzol™ Reagent (Invitrogen, Carlsbad, CA) for isolation according to the manufacturer's instructions. cDNA was synthesized with ReverTra Ace™ (Toyobo Co., Osaka, Japan) reverse transcriptase. RT-qPCR analysis was carried out via the use of a QuantStudio 3 Real-Time PCR System (Applied Biosystems, Foster City, CA) using TB Green® Premix Ex Taq™ II (Tli RNaseH Plus) (Takara Bio, Shiga, Japan) with 40 cycles at 95 °C for 15 s and annealing and extension at 60 °C for 1 min. Glyceraldehyde-3-phospate dehydrogenase (GAPDH) was used as a housekeeping gene, and the mRNA levels of each gene were normalized by the GAPDH mRNA levels. The primers using for RT-qPCR were purchased from Takara Bio. The sample size was set to n = 6 and this procedure was repeated three times in different sets.

### Statistical analysis

Statistical analyses were performed using the JMP 14.0. mRNA expression data between 6 groups were compared by Tukey–Kramer test. A *P* value of < 0.05 was considered statistically significant.

### Ethical approval

This study is reported according to the ARRIVE guidelines. Approval for all experimental procedures performed in this study was obtained from the Committee on Animal Research of Kyoto Prefectural University of Medicine, Kyoto, Japan. And all experiments were performed in accordance with the ethical guidelines of the ARVO Statement for the Use of Animals in Ophthalmic and Vision Research.

## Supplementary Information

Supplementary Information 1.

Supplementary Information 2.

Supplementary Information 3.

Supplementary Information 4.

## Data Availability

All data generated or analyzed during this study are included in this published article (and its Supplementary Information files).
