# Supplementary material for: Differences in gene regulation by TLR3 and IPS-1 signaling in murine corneal epithelial cells
Source: Sci Rep. 2023 May 16;13:7925. doi: 10.1038/s41598-023-35144-1 (PMC10188512; doi:10.1038/s41598-023-35144-1)
Supplement: Supplementary file 2 — Supplementary Information 2. [file 41598_2023_35144_MOESM2_ESM.pdf]

Supplementary Figure 1.

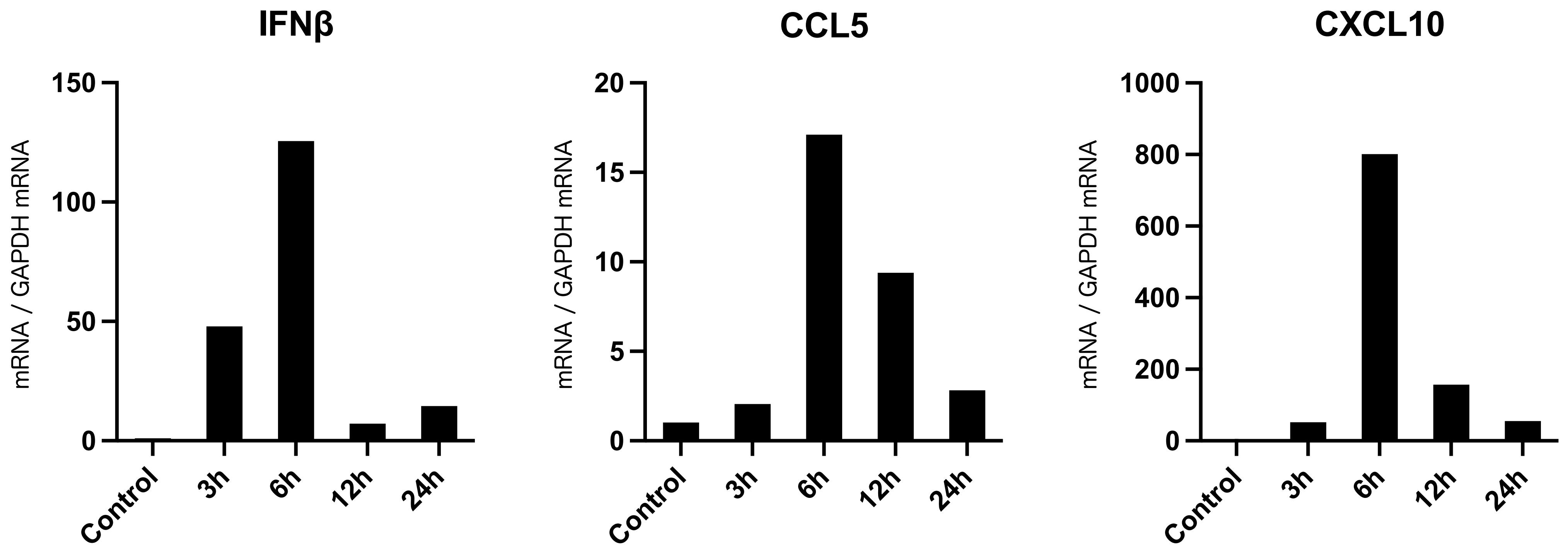

Supplementary Figure 1. 24-hour RT-qPCR gene expression in polyI:C stimulated primary cultured corneal epithelial cells from BALB/c wild-type mice. Corneal epithelial cells harvested from 4 eyes were mixed and cultured, then treated with 10 µg/ml polyI:C. Gene expression of IFNβ, CCL5, and CXCL10 was measured by RT-qPCR in unstimulated (control) cells; and at 3, 6, 12, and 24 hours in polyI:C stimulated cells. This graph presents data from an experiment that was repeated twice and produced the same results. The peak expression for all genes was at 6 hours.
