# Supplementary material for: Differences in gene regulation by TLR3 and IPS-1 signaling in murine corneal epithelial cells
Source: Sci Rep. 2023 May 16;13:7925. doi: 10.1038/s41598-023-35144-1 (PMC10188512; doi:10.1038/s41598-023-35144-1)
Supplement: Supplementary file 3 — Supplementary Information 3. [file 41598_2023_35144_MOESM3_ESM.docx]

**Supplementary Table 1.** Genes expressed more than twofold in WT murine primary cultivated corneal epithelial cells stimulated by PoIyI:C compared to WT without PolyI:C.

| Gene Symbol | | |
| --- | --- | --- |
| Cmpk2 | Xaf1 | Trafd1 |
| Ifit1 | Dhx58 | LOC105243966; Gm16026 |
| Rsad2 | Ifi203 | LOC102633783 |
| Ifit3 | Ifit1bl2 | Daxx |
| Ifit3b | Apol9a | Slfn4 |
| Cxcl10 | Ifit2 | Parp10; Plec |
| Mx2 | Herc6; 4930533I22Rik | Usp18 |
| Ch25h | Tlr2 | Pglyrp3 |
| Slfn2 | Zbp1 | Trim25 |
| Rnf225 | Apol9b | Rnf213 |
| Rtp4 | Trim30a | Mx1 |
| Oasl1 | Gbp2 | Trim14 |
| Ifi205 | Stat1 | Ifnl3 |
| Gbp3 | Ifih1 | Trim34b; Trim34a |
| Irgm1 | Tgtp1; Gm12185 | Rnf213 |
| Igtp; Irgm2 | Gbp2b; Gbp5 | Znfx1 |
| Irg1 | Dtx3l | Ifnl2 |
| Slfn5 | Gm7582 | Tap1 |
| Gm7609 | Oasl2 | Trav14d-3-dv8 |
| Ccrl2 | Gvin1; Gm4070 | Irf9 |
| Gm15433 | Tmem140 | Rnf213 |
| Slfn8 | Parp9 | Gm23941 |
| Csprs; Gm7609 | Neurl3 | Tnip3 |
| Trim21 | Oas3 | Rnf213 |
| Gm2666; Csprs | Slfn3 | H2-K2 |
| Ddx60 | Phf11d; Phf11c | Gm8989 |
| Gbp7 | Ddx58 | Mitd1 |
| Ifi47; Olfr56 | Mnda; Ifi204 | Casp4 |
| Ifi44l | Gbp4 | Rnf213 |
| Cd274 | Gm25794 | Lipg |
| Parp14 | Helz2 | n-R5s29 |
| Tnfsf10 | Gm23330 | Tlr3 |
| Il15 | Trim30d | Rnd1 |
| Ccl5 | AI607873 | Fam46a |
| Gbp9 | Sp100 | Gm23730 |
| Gm9706 | Lce3e | Parp12 |
| Gbp10; Gbp6 | Csf1 | Il6 |
| Oas2 | Tgtp2; Tgtp1 | Rnf213 |
| Samd9l | Eif2ak2 | Rnf213 |
| Irf1 | Lce3b |  |
| Stat2 | Icam1 |  |
