# Supplementary material for: Differences in gene regulation by TLR3 and IPS-1 signaling in murine corneal epithelial cells
Source: Sci Rep. 2023 May 16;13:7925. doi: 10.1038/s41598-023-35144-1 (PMC10188512; doi:10.1038/s41598-023-35144-1)
Supplement: Supplementary file 4 — Supplementary Information 4. [file 41598_2023_35144_MOESM4_ESM.docx]

**Supplementary Table 2.** Genes expressed less than 50% in TLR3KO or IPS-1KO murine primary cultivated corneal epithelial cells stimulated by PoIyI:C compared to WT with PolyI:C.

| WT vs TLR3KO  Gene symbol |  | WT vs IPS-1 KO  Gene symbol | | |
| --- | --- | --- | --- | --- |
| Irg1 |  | Mavs | Nlrc5 | Gm7582 |
| Cxcl10 |  | B4galnt2 | Bst2 | Klhl24 |
| Tmem72 |  | Car6 | Fam171b | Mir18 |
| Neurl3 |  | Tmem72 | Nlrc5 | Epdr1 |
| Fam171b |  | Gpnmb | Gbp10; Gbp6 | Nlrc5 |
| Rnd1 |  | Cxcl10 | Lcn2 | Jun |
| Rnf225 |  | Avil | Chchd10 | Pfpl |
| Pglyrp3 |  | Gvin1; Gm4070 | Acot2 | Ddit3 |
| Tlr2 |  | Gbp9 | Oas2 | Gm3402; Gm3415 |
| Cmpk2 |  | Dusp4 | Anpep | Nlrc5 |
| n-R5s29 |  | Gm15433 | Cxcl2 | Gabarapl1 |
| Mmp13 |  | Bend6 | Gm25328 | Dennd4a |
| Arl2bp |  | Csprs; Gm7609 | Slfn5 | Atg14 |
| Icam1 |  | Gm7609 | Nlrc5 | n-R5s29 |
| Gvin1; Gm4070 |  | Gm8989 | Mx2 | Faxc |
| Il15 |  | Gm2666; Csprs | Bhlhe41 | Tnfaip2 |
| Oasl1 |  | Ifi27l2a | Kctd4 | Dpp7 |
| Ccl5 |  | Gm14214 | Timp3 | Cep126 |
| Mmp1b |  | Serpinb6c | Icam1 | Slfn8 |
| Ifit3 |  | Gm8995 | Stmn2 | 4930555K19Rik |
| Fas |  | Gm8979; Gm8989 | Rnd1 | Slc2a2 |
| Rsad2 |  | Ly6a | Nlrc5 | Slc16a1 |
| Slfn2 |  | Gm29609 | Nlrc5 | Crebrf |
| Lipg |  | Dhrs9 | Zbtb10 | Myom2 |
| (No name) |  | Gm23287 | AA467197; Mir147 | Ifnl3 |
| Cxcl2 |  | Trim30a | Exph5 | Dennd4a |
| Gm25794 |  | Slfn2 | Irf7 | Ccl5 |
| Gbp3 |  | Soat2 | AI607873 | Dennd4a |
| Casp4 |  | Obox4-ps28 | Gm14056 | Atf3 |
| Csf1 |  | Pglyrp3 | Tbc1d9 | Hmcn1 |
| Nppb |  | Rragd | Gm25794 | Il6 |
| Car6 |  | Slfn4 | Nlrc5 | Nlrc5 |
| B4galnt2 |  | H2-K2 | Bmp6 | Eif5a2 |
| Ifit2 |  | Csf1 | Tlr2 | Dennd4a |
| Slfn8 |  | Ccl20 | Irg1 | (No name) |
| Gm8989 |  | Obox4-ps27 | Dennd4a | Olfr172 |
| Tslp |  | Acot5 | H2-Q8; H2-Q6 |  |
| Gbp9 |  | Casp4 | Dennd4a |  |
| Ccl20 |  | Obox4- ps33, 15, 18, 19, 20, 22, 21, 22 | |  |
| Cd274 |  | Pmaip1 |  |  |
| Ifit3b |  | Rpp25 |  |  |
| Gm8979; Gm8989 |  | Gla |  |  |
| Olfr1085 |  | Dennd4a |  |  |
